# Hsa_circ_0041150 serves as a novel biomarker for monitoring chemotherapy resistance in small cell lung cancer patients treated with a first-line chemotherapy regimen

**DOI:** 10.1007/s00432-023-05317-6

**Published:** 2023-08-28

**Authors:** Yang Zhang, Fengmei Chao, Lihua Lv, Ming Li, Zuojun Shen

**Affiliations:** 1 Cheeloo College of Medicine, Shandong University, Jinan, China; 2 The First Affiliated Hospital of USTC, Division of Life Sciences and Medicine, University of Science and Technology of China, Hefei, 230001 Anhui China; 3 Department of Laboratory Medicine, The First Affiliated Hospital of USTC, Division of Life Sciences and Medicine, University of Science and Technology of China, Hefei, 230031 Anhui China; 4 Core Unit of National Clinical Research Center for Laboratory Medicine, Hefei, China; 5 Division of Life Sciences and Medicine, Department of Cancer Epigenetics Program, The First Affiliated Hospital of USTC, University of Science and Technology of China, Hefei, 230001 Anhui China

**Keywords:** hsa_circ_0041150, Extracellular vesicles, Small cell lung cancer, Monitor chemoresistance

## Abstract

**Purpose:**

To explore the potential of circRNAs as biomarkers in non-invasive body fluids for monitoring chemotherapy resistance in SCLC patients.

**Methods:**

CircRNAs were screened and characterized using transcriptome sequencing, Sanger sequencing, actinomycin D treatment, and Ribonuclease R assay. Our study involved 174 participants, and serum samples were collected from all chemotherapy-resistant patients (n = 54) at two time points: stable disease and progressive disease. We isolated and identified serum extracellular vesicles (EVs) from the patients using ultracentrifugation, transmission electron microscopy, nanoflow cytometry, and western blotting analysis. The expression levels of serum and serum EVs circRNAs were determined by quantitative real-time polymerase chain reaction (qRT-PCR). The impact of circRNA on the function of SCLC cells was assessed through various assays, including proliferation assay, scratch assay, transwell assay, and cisplatin resistance assay.

**Results:**

Hsa_circ_0041150 was found to be upregulated in chemoresistant SCLC cells and played a role in promoting proliferation, invasion, migration, and cisplatin resistance. Furthermore, the expression levels of hsa_circ_0041150 in serum and serum EVs increased when SCLC patients developed resistance after a first-line chemotherapy regimen. When combined with NSE, the monitoring sensitivity (70.37%) and specificity (81.48%) for chemotherapy resistance significantly improved. Moreover, the expression level of hsa_circ_0041150 showed significant associations with time to progression from SD to PD, and high hsa_circ_0041150 levels after drug resistance were more likely to cause chemotherapy resistance. Additionally, hsa_circ_0041150 demonstrated valuable potential in monitoring the progression from initial diagnosis to chemotherapy resistance in SCLC patients.

**Conclusion:**

Thus, EVs hsa_circ_0041150 holds promise as a biomarker for monitoring chemotherapy resistance in SCLC patients.

**Supplementary Information:**

The online version contains supplementary material available at 10.1007/s00432-023-05317-6.

## Introduction

Based on Global Cancer Statistics 2020 (Sung et al. 2021), lung cancer remains the leading cause of cancer death, with an estimated 2.2 million new cases and 1.8 million deaths. The latest data (Cao et al. 2021; Wu et al. 2021) also indicates that lung cancer is the primary cause of cancer-related deaths for both males and females in China, accounting for 40% of global lung cancer mortalities, imposing a significant burden on society. Within the spectrum of lung cancers, small-cell lung cancer (SCLC) constitutes only 15% of cases; however, it poses a grave threat due to its remarkably high recurrence rate, strong propensity for early metastasis, and poor prognosis (Rudin et al. 2021). SCLC is an aggressive neuroendocrine cancer, closely linked to exposure to tobacco carcinogens (Bernhardt and Jalal 2016). Based on its developmental stage, SCLC is commonly categorized into two clinical staging classifications: limited-stage (LD) and extensive stage (ED) (Dingemans et al. 2021). Due to its rapid tumor growth and early metastatic dissemination, more than two-thirds of patients are diagnosed with ED (Zugazagoitia and Paz-Ares 2022). Consequently, unlike the majority of solid tumors, surgery is not the preferred treatment for small cell lung cancer. Instead, the generally accepted first-line therapeutic approach involves a combined chemotherapy regimen containing platinum agents (Ganti et al. 2021).

The clinical challenge in treating SCLC lies in the rapid development of chemotherapy resistance, leading to tumor recurrence and distant metastasis (Zhu et al. 2022). However, obtaining tissue samples from SCLC patients is difficult as they typically do not undergo surgery, hindering the study of SCLC chemoresistance. Hence, there is a pressing need to explore novel biomarkers for SCLC chemoresistance from easily accessible samples.

CircRNAs were first discovered in the 1970s and were initially considered nonfunctional RNA splicing products with a closed-loop structure formed by head–tail covalent bond formation (Sanger et al. 1976). In recent years, the rapid progress in biological detection technology and bioinformatics has led to the identification of many circRNAs with varying lengths and types (Huang et al. 2020a; Wang et al. 2020). Consequently, circRNAs have emerged as a new research hotspot due to their potential involvement in the carcinogenic or anticancer pathways of tumors (Kristensen et al. 2022; Ghafouri-Fard et al. 2021). A growing body of evidence highlights the significant roles of circRNAs in tumorigenesis, chemoresistance, metastasis, and prognosis (Chen et al 2021; Gao et al 2021; Tang et al. 2021; Frey et al. 2021).

Recent studies have predominantly concentrated on investigating the role of circRNAs in platinum chemotherapy resistance in non-small cell lung cancer (NSCLC) (Zhu et al. 2020; Ren et al. 2022), with limited attention given to small-cell lung cancer (SCLC) (Huang et al. 2020b). CircRNAs exhibit favourable characteristics as potential biomarkers, owing to their inherent stability, and further exploration and research in the clinical application domain will provide crucial molecular insights (Yang et al. 2021a). Given the challenges of acquiring repeated tissue samples from cancer patients, there is growing interest among researchers regarding the feasibility of detecting circRNAs in alternative samples as promising noninvasive biomarkers for lung cancer (Hua et al. 2022).

Extracellular vesicles (EVs) are nanovesicles secreted by cells, with an average diameter of ~ 200 nm. They comprise diverse constituents, including nucleic acids, proteins, lipids, amino acids, and metabolites (Théry et al. 2018). EVs function as a cell-to-cell transit system within the human body, facilitating efficient exchange of cellular components. Detecting EVs in biological fluids has the potential to provide valuable diagnostic information, reflecting the changing states of cells or tissues (Mashouri et al. 2019). Current insights into EVs indicate that these nanovesicles and their components hold promise as novel biomarkers for diagnosing, prognosing, and therapeutically targeting lung cancer (Amiri et al. 2021). However, the mechanism of chemotherapy resistance in small-cell lung cancer (SCLC) remains poorly understood due to limited access to clinical samples.

Therefore, the key to unlocking this mystery lies in gaining access to clinical samples. Our research group has successfully identified miRNAs in exosomes as noninvasive biomarkers for the early diagnosis of lung adenocarcinoma (LUAD) (Wu et al. 2022) and promising therapeutic targets for SCLC chemoresistance (Li et al. 2021). However, as circRNAs can influence the expression of target mRNAs by binding to miRNAs (Yang et al. 2021b), our subsequent investigation has focused on the roles of EV circRNAs in SCLC chemoresistance.

During this study, we pinpointed the six most significantly upregulated circRNAs in SCLC chemoresistance and validated their expression levels in the serum and serum EVs of SCLC chemoresistant patients. Our findings strongly indicate that hsa_circ_0041150, present in both serum and serum EVs, holds substantial promise as a valuable biomarker for monitoring chemoresistance in SCLC patients undergoing first-line chemotherapy. By comparing the diagnostic performance and correlation with the clinical manifestation of circRNAs, we have established the potential of hsa_circ_0041150 as a novel noninvasive biomarker for monitoring chemoresistance in SCLC. This discovery may pave the way for exploring new and improved therapeutic strategies for treating SCLC.

## Materials and methods

### Cell culture

We purchased the SBC-3, and H446 cell lines from the Cell Bank of Type Culture Collection of the Chinese Academy of Sciences (Shanghai, China). The human SCLC cell lines H69, H82, SHP77, DMS273 were kept in our own laboratory. All cell lines were cultured in RPMI 1640 medium supplemented with 10% fetal bovine serum (Gibco, Australia) at 37 °C in a 5% CO2 incubator.

### Characteristics of circRNAs

We designed and synthesized the primers for circRNAs (Supplementary Table S2) through RiboBio (Guangzhou, China). Additionally, the primers for mRNAs were synthesized by Sangon Biotech (Shanghai, China). To verify the specific back splicing sites, we sent the amplification products of circRNAs to Sangon Biotech for Sanger sequencing.

To assess the stability of circRNAs, we detected the expression levels of circRNAs and their parent gene mRNAs at six different time points (0 h, 2 h, 4 h, 8 h, 12 h, 24 h) after treating the cells with actinomycin D (Aladdin, Shanghai, China). We compared the degree and rate of inhibition of circRNAs and mRNAs to evaluate the stability of circRNAs.

Furthermore, we used Ribonuclease R (RNase R), a ribonuclease that efficiently hydrolyzes linear RNA, to examine the stability of the structure. The RNA from cells was extracted and treated with or without RNase R (Epicenter, USA) while being placed on ice. After incubation at 37 °C for 30 min and subsequent incubation at 70 °C for 10 min, we detected the expression levels of circRNAs and mRNAs to compare the stability of the structure.

### Collection of SCLC patient serum samples

The clinical serum samples used in this study were collected from SCLC patients who underwent a first-line chemotherapy regimen at the West District of the First Affiliated Hospital of University of Science and Technology of China from July 2021 to October 2022. The study received approval from the Ethics Committee of West District of the First Affiliated Hospital of University of Science and Technology of China (No: Number 70 in 2021).

A total of 174 participants were recruited and categorized into three groups: chemotherapy-resistant patients (n = 54), patients at initial diagnosis (n = 60), and healthy controls (n = 60). The healthy controls consisted of hospital employees with no underlying diseases, and their liver and kidney function, as well as tumor markers, were within the normal range. We gathered clinical and pathological data from the healthy controls, including their age (ranging from 23 to 72 years old), sex (32 male and 28 female patients). For the patients at the initial diagnosis stage, they were diagnosed with SCLC without any prior clinical treatment. We collected their clinical and pathological data, including age (ranging from 48 to 81 years old), sex (50 male and 10 female patients).

The chemotherapy-resistant patients (n = 54) were all treated with the same platinum-based first-line chemotherapy regimen, and clinical manifestations and imaging examinations were used to evaluated all patients every two cycles (Kalemkerian et al. 2018). Their treatment efficacy was evaluated according to the respond evaluation criteria in solid tumors 1.1 (RECIST 1.1) and their disease states were categorized as complete response (CR), partial response (PR), stable disease (SD), or progressive disease (PD) (Eisenhauer et al. 2009). The time to progression after chemotherapy of the 54 patients were greater than 90 days. Serum samples were collected from the patients during each of these disease states (CR, PR, SD, and PD) to perform matching detection and comparison between the SD (chemosensitive) and PD (chemoresistant) statuses. Clinical and pathological data, including age (ranging from 40 to 82 years old), sex (38 male and 16 female patients), clinical stage (16 patients with LD and 38 patients with ED), time to develop resistance from SD to PD (ranging from 1 to 31 months), and whether they had lymphatic metastasis, distant metastasis, and smoking history, were also collected for these patients. Fortunately, we were able to collect an additional serum sample from 21 of these patients at the time of their initial diagnosis.

### Isolation of serum extracellular vesicles

The ultracentrifugation method was employed to isolate extracellular vesicles (EVs) from the serum of patients after peripheral blood collection. The process involved several steps: initially, serum and cells were separated by centrifugation at 3000×*g* for 10 min using separation gel tubes. Subsequently, the mixture was centrifuged again at 4 °C and 16,000×*g* for 45 min to eliminate cell debris and large shedding vesicles. The resulting supernatant was subjected to ultracentrifugation at 4 °C and 100,000 × g for 1 h using a Beckman Coulter Optima MAX-XP ultracentrifuge with a TLA55 rotor. After this step, the supernatant was carefully discarded, and the obtained pellets were resuspended in 1 ml of PBS. The resuspended mixture was subjected to another round of ultracentrifugation at 4 °C and 100,000×*g* for 1 h. Finally, the supernatant was discarded, and the isolated exosomes were resuspended in 20 μl of PBS for future use.

### Characterization and quantification of serum extracellular vesicles

The serum EVs were first fixed with a glutaraldehyde solution and then stained with 2% phosphotungstic acid at room temperature for 5 min. The morphologies of the EVs were observed using transmission electron microscopy (UltraScan 1000 CCD, FEI, USA) at an acceleration voltage of 120 keV.

The diameter and concentration of serum EVs were analysed using nanoflow cytometry (Fuliu Biotechnology, Xiamen, China). Serum EV specimens were combined with nanopore diameters of grains, which were mixed and labelled. The mixture was then detected by a nanoflow detector at an excitation wavelength of 532 nm using the N30 sample mixing system. The number of serum EVs was calculated based on the standard working curve size of the diameter.

To extract the proteins of serum EVs, protein lysis buffer was used, and samples were separated by 10% sodium dodecyl sulfate polyacrylamide gels and then electrotransferred onto polyvinylidene fluoride (PVDF) membranes. The membrane was blocked with 5% skim milk powder at room temperature for 2 h, and specific antibodies against human CD63 (1:1000, Proteintech, #25682-1-AP), TSG101 (1:1000, Novus, #NBP1-80659), and Calnexin (1:2000, Proteintech, #66903-1-Ig) were used for incubation. Subsequently, the membranes were incubated with protein-specific horseradish peroxidase-conjugated secondary antibodies (Beyotime, Shanghai, China) for 1 h. Finally, the band intensities were quantified using an imaging system (Tanon, Shanghai, China).

### RNA extraction and quantitative real-time PCR

Total RNA was extracted from cells, serum, and serum EVs using TRIzol reagent (Invitrogen, USA) following the manufacturer’s instructions. For reverse transcription PCR, 1 μg of total RNA was used to synthesize cDNA using HiScript^®^ II Reverse Transcriptase (Vazyme, Nanjing, China). Subsequently, 1 ng of cDNA was used for quantitative real-time PCR (qRT-PCR) with 2 × RealStar Fast SYBR qPCR Mix (GenStar, Beijing, China) on a LightCycler480 instrument (Roche, Germany).

The amplification conditions were set up as follows: 95 °C for 2 min, followed by 40 cycles of denaturation at 95 °C for 15 s, annealing at 60 °C for 40 s, and extension at 72 °C for 30 s. The internal control used was 18S ribosomal RNA (18S rRNA), and the comparative cycle threshold method 2 ^(−ΔΔCt)^ was employed to calculate the relative expression levels for data analysis.

### Detection of traditional tumor markers

Peripheral venous blood samples were collected from all chemotherapy-resistant patients at the time of stable disease (SD) and progressive disease (PD). The samples were then centrifuged at 3000 × g for 10 min to separate the serum. Tumor markers, including neuron-specific enolase (NSE), pro-gastrin-releasing peptide (ProGRP), carbohydrate antigen 125 (CA125), and cytokeratins 21–1 (CA211), were detected using an Alinty I electrochemiluminescence immunoanalyzer (Abbott, USA). All procedures were strictly conducted following the instructions of the reagents and instruments. The criteria for judgment were as follows: results were considered positive if they were above the upper limit of the normal reference value range [NSE: (0–16.3) ng/ml, ProGRP: (28.3–74.4) pg/ml, CA125: (0–35) U/ml, CA211: (0–3.3) ng/ml], and the calibration curves and quality control results were normal.

### Construction of stable cell lines

The hsa_circ_0041150 overexpression lentivirus and the corresponding negative control were obtained from Jisai (Guangzhou, China) and infected into H446 cells. The stably transfected cells were selected using puromycin (5 μg/ml, Sigma-Aldrich, St. Louis, MO, USA) to create the H446-overexpression cell line. The hsa_circ_0041150 siRNA and shRNA (Supplementary Table S4) were purchased from RiboBio (Guangzhou, China), and hsa_circ_0041150 knockdown lentiviruses and their corresponding negative controls were obtained from GeneChem (Shanghai, China). The hsa_circ_0041150 knockdown lentivirus was used to separately infect H446 and SHP77 cells. After stable transfection, the cells were selected using puromycin (5 μg/ml, Sigma-Aldrich, St. Louis, MO, USA) to establish the H446-knockdown cell line and SHP77-knockdown cell line. All procedures were strictly conducted following the instructions of the reagents and instruments.

### Cell proliferation assay

H446 cells (2000 cells per well) and SHP77 cells (3000 cells per well) were seeded in 96-well plates during the active growth phase. Once the cells were attached, 100 µl of 10% CCK-8 reagent (Beyotime, Shanghai, China) was added to each well. The plates were then incubated in a 37 °C, 5% CO2 incubator for 2 h. The absorbance was measured at 450 nm, and this process was repeated at the same time point for five consecutive days.

### Cell scratch assay

For the hsa_circ_0041150 overexpression and knockdown H446 cells (5 × 10^5^ to 1 × 10^6^ cells per well), 24-well plates were used during the active growth phase. A horizontal line was marked at the bottom of the wells, and the plates were incubated in a 37 °C, 5% CO2 incubator. The next day, a sterile pipette was used to create a vertical scratch, forming a wound. Images of the wounds were captured at 0 h, and then the medium was replaced, and the cells were further cultured in the incubator. After 30 h, the 24-well plate was removed, and the wounds were observed and photographed under a microscope to analyze the invasion ability of the different cells.

### Cell transwell assay

Subsequently, 800 µl of medium containing 10% FBS and a Transwell chamber (Corning, USA) were added to 24-well plates. The hsa_circ_0041150 overexpression and knockdown H446 cells (5 × 10^4^ to 1 × 10^5^ cells per well) were seeded in the Transwell chambers during the active growth phase and suspended in medium. The cells were then placed in a 5% CO2 incubator at 37 °C, and after 48 h, the upper chambers were removed. The bottom of the chambers was fixed with 4% paraformaldehyde (Beyotime, Shanghai, China) for 15 min and stained with 0.1% crystal violet solution (Beyotime, Shanghai, China) for 20 min at room temperature. Subsequently, the Transwell chambers were observed and photographed under a microscope, and the migration ability of the different cells was quantified and analyzed.

### Cisplatin resistance assay

H446 cells (3000 cells per well) were seeded in 96-well plates during the active growth phase. Cisplatin (Solarbio, Beijing, China) was dissolved in DMF (N′,N-Dimethylformamide) (Solarbio, Beijing, China) and added to the medium to achieve final concentrations of 50, 100, 150, 200, and 250 μg/ml, respectively. Once the cells adhered, 100 µl of medium containing different concentrations of cisplatin (50, 100, 150, 200, 250 μg/ml) were added to the 96-well plate and incubated at 37 °C and 5% CO_2_ in the incubator. After 24 h, 100 µl of 10% CCK-8 reagent (Beyotime, Shanghai, China) was added to the 96-well plate and incubated at 37 °C and 5% CO_2_ for 2 h. The absorbance was then measured at 450 nm to assess cell viability.

### Statistical analysis

Statistical analysis was conducted using GraphPad Prism 8.0 (Graph Pad Software, CA, USA) and SPSS 25.0 (IBM Corp, Armonk, NY, USA). ImageJ software (National Institutes of Health, USA) was employed to analyze the cell scratches and migrating cells among different groups. After performing the Kolmogorov–Smirnov test, normally distributed data were expressed as x ± s, while non-normally distributed data were represented as the median (interquartile range). For data analysis and comparison between different groups, the t-test and non-parametric tests (Mann–Whitney U and Kruskal–Wallis H rank sum) were utilized based on the nature of the data. Receiver operating characteristic (ROC) curve analysis was employed to obtain the area under the curve (AUC) for each marker. The corresponding sensitivity and specificity were calculated using the largest Youden index (sensitivity + specificity − 1). To facilitate statistical analysis, the (2^−ΔΔCt^) results were multiplied by 10^3^ and used to determine the circRNA expression levels in serum and serum EVs. The differences in clinicopathological variables between the hsa_circ_0041150-high and hsa_circ_0041150-low groups were analyzed using the chi-square test. The two groups were evaluated using the Kaplan–Meier method, and a p value of < 0.05 was considered statistically significant.

## Results

### Six circRNAs were stable and upregulated significantly in chemoresistant SCLC cells

**Fig. 1:**
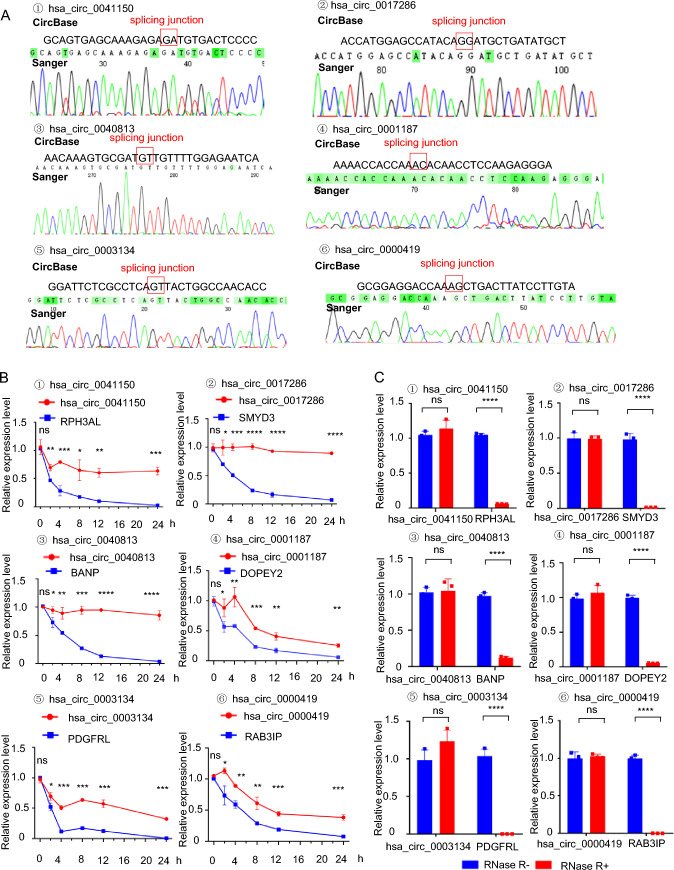
Upregulated circRNAs were stable.** a** The cyclization sites of circRNAs were identified using Sanger sequencing. The sequences in circBase (upper panel) were found to be in agreement with the results obtained from Sanger sequencing (lower panel). The red box highlights the back-splicing site of the circRNAs. **b** CircRNAs were more stable than linear transcripts after treatment with actinomycin D, ****p < 0.0001, ***p < 0.001, **p < 0.01, *p < 0.05, ns: no statistically significant difference. **c** CircRNAs were more tolerant to digestion by RNase R enzymes than linear transcripts, ****p < 0.0001, ns: no statistically significant difference

**Fig. 2:**
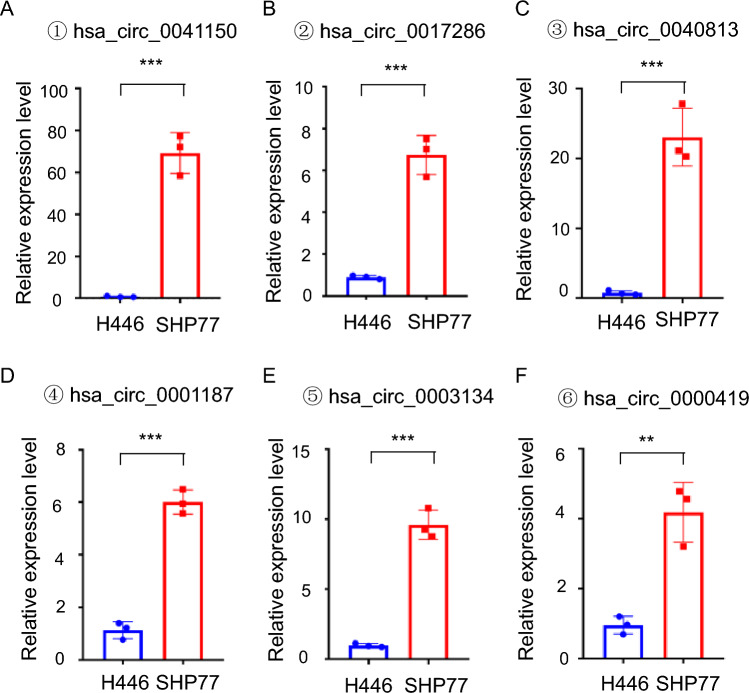
The expression levels of circRNAs in SHP77 cells were significantly higher than those in H446 cells. **a** The expression level of hsa_circ_0041150 in SHP77 cells was higher than H446 cells, ***p < 0.001. **b** The expression level of hsa_circ_0017286 in SHP77 cells was higher than H446 cells, ***p < 0.001. **c** The expression level of hsa_circ_0040813 in SHP77 cells was higher than H446 cells, ***p < 0.001. **d** The expression level of hsa_circ_0001187 in SHP77 cells was higher than H446 cells, ***p < 0.001. **e** The expression level of hsa_circ_0003134 in SHP77 cells was higher than H446 cells, ***p < 0.001. **f** The expression level of hsa_circ_0000419 in SHP77 cells was higher than H446 cells, **p < 0.01

Based on previous work (Chao et al. 2023), we identified the chemoresistant cell line (SHP77) and chemosensitive cell line (H446) from six SCLC cell lines, including H69, H82, H446, SHP77, SBC-3, and DMS273, based on the relative IC50 (the drug dose required for 50% of cells killed) and resistance index of the first-line chemotherapy drugs of SCLC, such as cisplatin (DDP), etoposide (VP-16), and adriamycin (ADM). Transcriptome high-throughput sequencing of the H446 and SHP77 cell lines revealed 1181 differentially expressed circRNAs (the NGS data GSE193854 can be found on the National Center for Biotechnology Information website). Among these, 8 circRNAs were upregulated, and 5 circRNAs were downregulated, meeting the criteria of |log_2_Fold Change|> 4 and p < 0.05 (Supplementary Figure S1). We then focused on the upregulated circular RNAs (Supplementary Table S1) to identify novel biomarkers for diagnosing drug resistance in SCLC. Subsequently, the upregulated circRNAs were validated through qRT-PCR. The sequences of the six circRNAs (hsa_circ_0041150, hsa_circ_0017286, hsa_circ_0040813, hsa_circ_0001187) in circBase were consistent with the results of Sanger sequencing (Fig. 1a). Additionally, these six circRNAs exhibited higher stability than linear transcripts after treatment with actinomycin D (Fig. 1b) and were resistant to RNase R digestion (Fig. 1c), supporting their circular forms. Moreover, the expression levels of these six circRNAs in SHP77 cells were significantly higher than those in H446 cells, consistent with the sequencing results (Fig. 2a–f).

### The expression levels of these six circRNAs were upregulated in the serum of SCLC chemoresistant patients

**Fig. 3:**
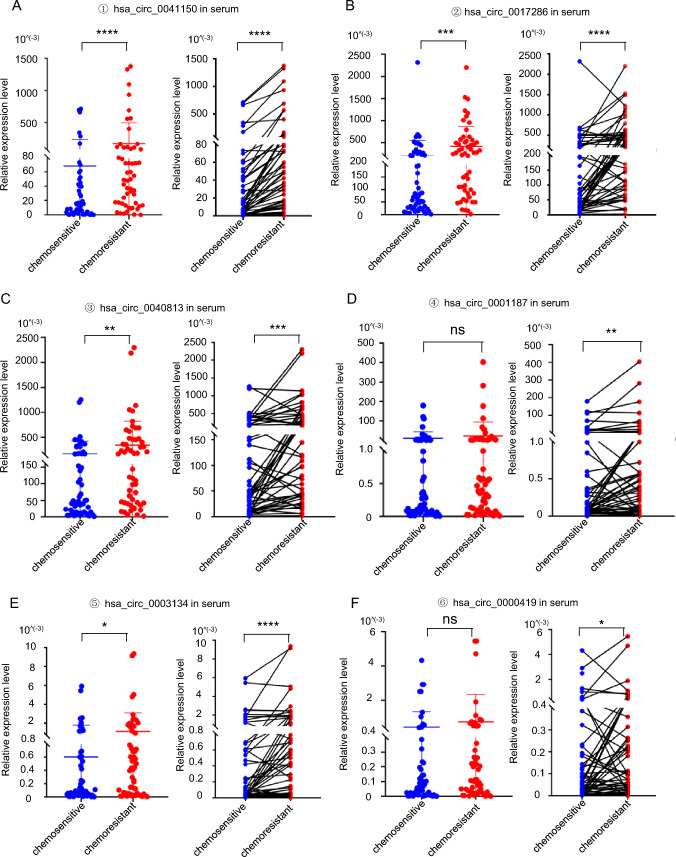
The expression levels of circRNAs were upregulated in the serum of SCLC chemoresistant patients. All the results are shown with groups (left) and pairings (right), n = 54. **a** The expression level of hsa_circ_0041150 was found to be upregulated in the serum of patients with chemoresistant small cell lung cancer (SCLC), ****p < 0.0001. **b** The expression level of hsa_circ_0017286 was found to be upregulated in the serum of patients with chemoresistant small cell lung cancer (SCLC), ****p < 0.0001, ***p < 0.001. **c** The expression level of hsa_circ_0040813 was found to be upregulated in the serum of patients with chemoresistant small cell lung cancer (SCLC), ***p < 0.001, **p < 0.01. **d** The expression level of hsa_circ_0001187 was found to be upregulated in the serum of patients with chemoresistant small cell lung cancer (SCLC), **p < 0.01, ns: no statistically significant difference. **e** The expression level of hsa_circ_0003134 was found to be upregulated in the serum of patients with chemoresistant small cell lung cancer (SCLC), ****p < 0.0001, *p < 0.05. **f** The expression level of hsa_circ_0000419 was found to be upregulated in the serum of patients with chemoresistant small cell lung cancer (SCLC), *p < 0.05, ns: no statistically significant difference

To assess the clinical significance of these six circular RNAs in patients with chemotherapy-resistant SCLC, we collected serum samples from the same patients who underwent first-line chemotherapy at both the time points of stable disease (SD) and progressive disease (PD) as paired samples. Our analysis revealed that four circRNAs (hsa_circ_0041150, hsa_circ_0017286, hsa_circ_0040813, and hsa_circ_0003134) exhibited an increase after the development of chemoresistance (Fig. 3a–f, left). Moreover, when comparing the serum levels of all six circRNAs between the time points of chemotherapy resistance (PD) and stable disease (SD) in the same patient (n = 54), we observed a significant elevation in their expression during chemotherapy resistance (Fig. 3a–f, right).

### The expression levels of these six circRNAs were upregulated in serum extracellular vesicles (EVs) of SCLC chemoresistant patients

**Fig. 4:**
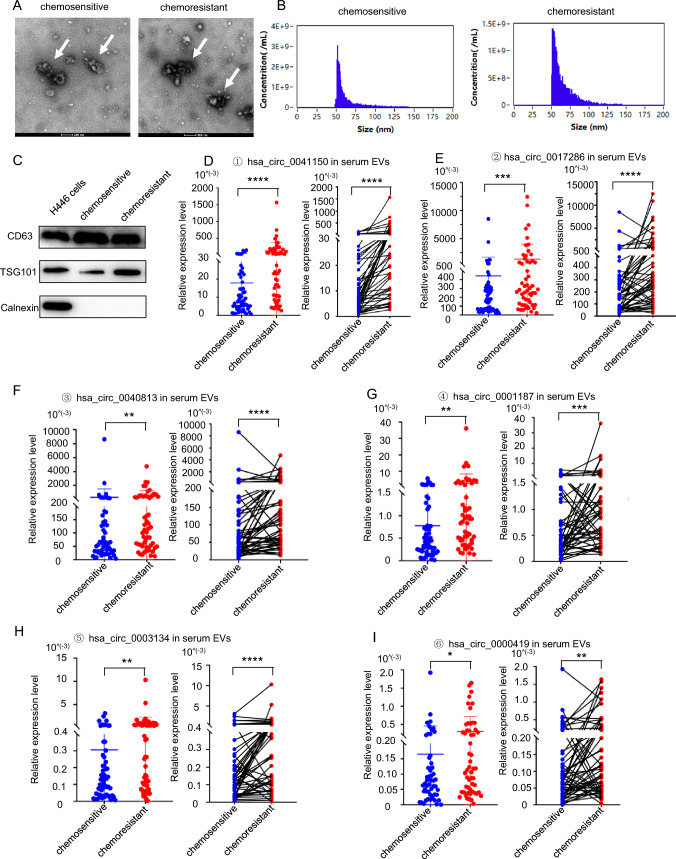
The expression levels of circRNAs were upregulated in serum extracellular vesicles (EVs) of SCLC chemoresistant patients. **a** The morphology and size of serum EVs were observed by transmission electron microscopy. Scale bar, 200 nm. **b** The concentration, particle size and diameter distribution of serum EVs were analyzed by nanoflow assay. **c** The expression of the EV iconic protein markers CD63 and TSG101 in serum EVs was detected by western blotting. **d** The expression level of hsa_circ_0041150 was upregulated in serum EVs of SCLC chemoresistant patients, ****p < 0.0001. **e** The expression level of hsa_circ_0017286 was upregulated in serum EVs of SCLC chemoresistant patients, ****p < 0.0001, ***p < 0.001. **f** The expression level of hsa_circ_0040813 was upregulated in serum EVs of SCLC chemoresistant patients, ****p < 0.0001, **p < 0.01. **g** The expression level of hsa_circ_0001187 was upregulated in serum EVs of SCLC chemoresistant patients, **p < 0.01, ***p < 0.001. **h** The expression level of hsa_circ_0003134 was upregulated in serum EVs of SCLC chemoresistant patients, ****p < 0.0001, **p < 0.01. **i** The expression level of hsa_circ_0000419 was upregulated in serum EVs of SCLC chemoresistant patients, *p < 0.05, **p < 0.01

We employed ultracentrifugation to isolate EVs from serum samples, aiming to investigate whether these six circRNAs were specifically contained within EVs. The characterization of serum EVs was conducted through transmission electron microscopy (TEM), nanoflow cytometry (NanoFCM), and western blotting to assess their morphology, concentration, and markers. TEM analysis revealed the presence of a typical visible membrane bilayer with a size smaller than 200 nm (Fig. 4a). The NanoFCM results demonstrated a nanoparticle concentration of (2.03–2.53) × 10^10^ particles/ml, and the particle size of EVs ranged from 50 to 200 nm (Fig. 4b). Western blotting further confirmed the presence of classical EV protein markers CD63 and TSG101 (Fig. 4c), indicating the successful extraction of EVs from clinical serum samples. Following this, we examined the expression of the six circRNAs in serum EVs after the development of drug resistance (Fig. 4d–i, left). The results showed an increase in all six circRNAs in serum EVs after chemotherapy resistance. Moreover, the expression levels of these circRNAs were significantly higher in the serum EVs of chemotherapy-resistant patients compared to those at the time of stable disease in the same patient (n = 54) (Fig. 4d–i, right). Notably, our findings highlighted the superior performance of serum EVs in predicting chemotherapy resistance compared to serum alone, with hsa_circ_0041150 exhibiting the most significant difference after chemotherapy resistance in both serum and serum EVs compared with other circRNAs.

### The hsa_circ_0041150 had better diagnostic efficiency for chemotherapy resistance in SCLC patients

Hsa_circ_0041150 is an exonic circRNA and located chr17:131558-177370 with the size of 474 bp.

**Fig. 5:**
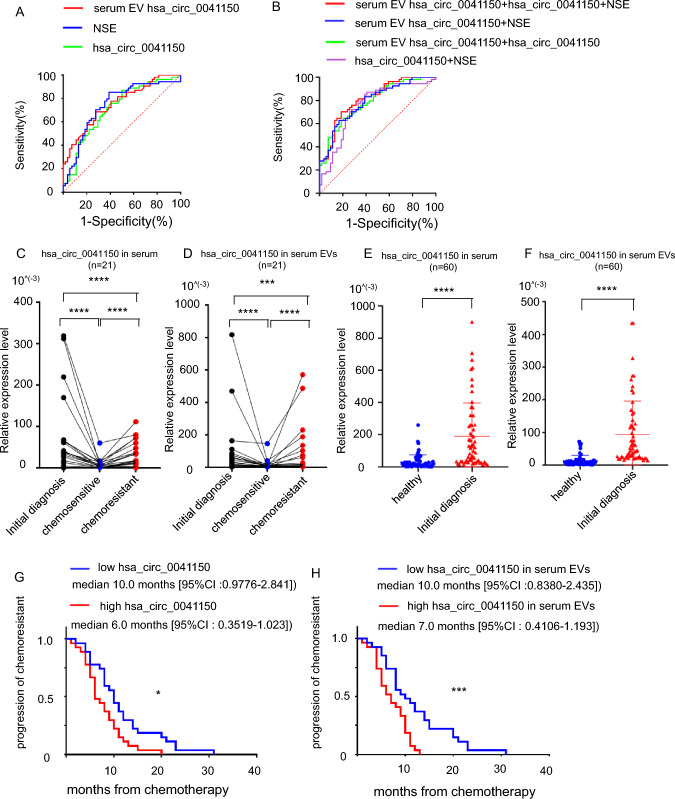
Hsa_circ_0041150 had good value for monitoring chemotherapy resistance in SCLC patients. **a** The diagnostic efficiency of serum hsa_circ_0041150 (AUC: 0.7145) and serum EV hsa_circ_0041150 (AUC: 0.7531) and NSE (AUC: 0.7414) in SCLC chemoresistant patients. **b** The combined diagnostic efficiency of serum EV hsa_circ_0041150 + hsa_circ_0041150 + NSE (AUC: 0.8148) had advantages over other combinations. **c** The serum hsa_circ_0041150 level can be used to monitor the progression of chemotherapy resistance in SCLC patients, ****p < 0.0001. **d** The serum EV hsa_circ_0041150 level can be used to monitor the progression of chemotherapy resistance in SCLC patients, ****p < 0.0001, ***p < 0.001. **e** The serum hsa_circ_0041150 level in SCLC patients whowere initially diagnosed was significantly higher than that in healthy controls, ****p < 0.0001. **f** The serum EV hsa_circ_0041150 level in SCLC patients who were initially diagnosed was significantly higher than that in healthy controls, ****p < 0.0001. **g** SCLC patients with high serum hsa_circ_0041150 levels after drug resistance were more likely to experience chemotherapy resistance, *p < 0.05. **h** SCLC patients with high serum EV hsa_circ_0041150 levels after drug resistance were more likely to experience chemotherapy resistance, ***p < 0.001

To further explore the diagnostic value of hsa_circ_0041150 in chemotherapy resistance, we assessed the expression levels and areas under the ROC curves (AUCs) of six circRNAs in both serum and serum EVs. By comparing the diagnostic efficacy of serum circRNAs, EV circRNAs, and traditional clinical tumor markers for lung cancer, namely NSE, proGRP, CA125, and CA211 (Table 1 and Supplementary Table S3), we observed that hsa_circ_0041150 in EVs (AUC: 0.7531) (Supplementary Figure S2a) and in serum (AUC: 0.7145) (Supplementary Figure S2b) demonstrated greater diagnostic value for chemotherapy resistance compared to other circRNAs. Notably, NSE (AUC: 0.7414) outperformed other clinical tumor markers in predicting chemotherapy resistance in SCLC patients (Supplementary Figure S2c). Among these three markers, EV hsa_circ_0041150 exhibited a more robust predictive effect for chemotherapy-resistant SCLC patients (Fig. 5a). Moreover, the combination of these three markers showed even better performance (AUC: 0.8148) than any single indicator (Fig. 5b), with increased sensitivity (70.37%) and specificity (81.48%) (Table 1).

**Table 1:** Hsa_circ_0041150 combined with NSE can improve the diagnostic sensitivity and specificity of SCLC chemoresistant patients

Markers	AUC (95% CI)	Sensitivity (%)	Specificity (%)	p value
hsa_circ_0041150	0.7145 (0.6168, 0.8122)	72.22	62.96	*0.0001
Serum EV hsa_circ_0041150	0.7531 (0.6633, 0.8429)	68.52	72.22	* < 0.0001
NSE	0.7414 (0.6454, 0.8375)	70.37	72.22	* < 0.0001
hsa_circ_0041150 + NSE	0.7551 (0.6614, 0.8489)	72.22	72.22	* < 0.0001
Serum EV hsa_circ_0041150 + hsa_circ_0041150	0.7898 (0.7064, 0.8732)	62.96	81.48	* < 0.0001
Serum EV hsa_circ_0041150 + NSE	0.7905 (0.7068, 0.8741)	83.33	61.11	* < 0.0001
Serum EV hsa_circ_0041150 + hsa_circ_0041150 + NSE	0.8148 (0.7360, 0.8936)	70.37	81.48	* < 0.0001

*P < 0.05

### Hsa_circ_0041150 had good value in monitoring the progression from initial diagnosis to chemotherapy resistance in SCLC patients

We conducted a focused analysis on 21 SCLC patients who underwent first-line chemotherapy at our hospital. We compared the levels of hsa_circ_0041150 in both serum and serum EVs at three different time points: initial diagnosis, stable disease (SD), and progressive disease (PD). Interestingly, we observed that hsa_circ_0041150 was initially at a high level in both serum and serum EVs when patients were diagnosed with SCLC without any prior treatment. However, after the effective administration of the first-line chemotherapy regimen, the expression levels of hsa_circ_0041150 in both serum and serum EVs rapidly decreased, showing a significant difference (p < 0.0001). Subsequently, during the development of chemotherapy resistance and clinical progression, the levels of hsa_circ_0041150 in both serum and serum EVs significantly increased once again (p < 0.0001) (Fig. 5c–d). To further investigate the potential role of hsa_circ_0041150 in monitoring the disease progression of SCLC, we also studied 60 healthy individuals from our hospital as the control group. Additionally, we examined the expression levels of hsa_circ_0041150 in another 60 newly diagnosed SCLC patients. The levels of hsa_circ_0041150 in both serum and serum EVs were found to be significantly higher in the newly diagnosed SCLC patients compared to the healthy control group (Fig. 5e–f). These findings suggest that the level of hsa_circ_0041150 could serve as a useful indicator to monitor the disease progression and chemotherapy resistance in SCLC.

### The cinlincal association between hsa_circ_0041150 expression levels and clinicopathological features in SCLC chemoresistant patients

Based on the median value of the serum and serum EV hsa_circ_0041150 expression levels, chemotherapy-resistant SCLC patients were categorized into two groups: hsa_circ_0041150-low and hsa_circ_0041150-high groups. We conducted an analysis to investigate the association between hsa_circ_0041150 expression levels and various clinicopathological parameters, including age, sex, clinical stage, lymphatic metastasis, distant metastasis, smoking history, and time to progression from SD to PD. The results indicated a significant correlation between hsa_circ_0041150 expression levels and time to progression from SD to PD (Table 2). And the results revealed that SCLC patients with high hsa_circ_0041150 levels in both serum and serum EVs after developing drug resistance were more prone to experience chemotherapy resistance (Fig. 5g–h). This observation suggests the potential involvement of hsa_circ_0041150 in the progression of chemoresistance, warranting further investigation and exploration of its precise role in this context.

**Table 2:** The clinical characteristics of hsa_circ_0041150 in serum and serum EVs of SCLC chemoresistant patients

Variable	serum hsa_circ_0041150 level relative expression × 10^3^ (median = 49.38)		χ^2^ value	p value	serum EV hsa_circ_0041150 level relative expression × 10^3^ (median = 24.35)		χ^2^ value	p value
	Low (n = 27)	High (n = 27)			Low (n = 27)	High (n = 27)		
Age (years)								
< 60.5	12	15	0.667	0.414	15	12	0.667	0.414
≥ 60.5	15	12			12	15		
Gender								
Male	19	19	0.001	0.999	19	19	0.001	0.999
Female	8	8			8	8		
Clinical stages								
LD	11	5	3.197	0.074	6	10	1.421	0.233
ED	16	22			21	17		
Lymphatic metastasis								
No	4	9	2.533	0.112	4	9	2.533	0.112
Yes	23	18			23	18		
Distant metastasis								
No	11	7	1.333	0.248	7	11	1.333	0.248
Yes	16	20			20	16		
Smoking history								
No	17	20	0.773	0.379	18	19	0.086	0.293
Yes	10	7			9	8		
Time to progression from SD to PD (months)								
< 8.0	7	15	4.909	*0.027	7	15	4.909	*0.027
≥ 8.0	20	12			20	12		

*P < 0.05

### The hsa_circ_0041150 promoted the proliferation, invasion, migration and cisplatin resistance of SCLC cells

**Fig. 6:**
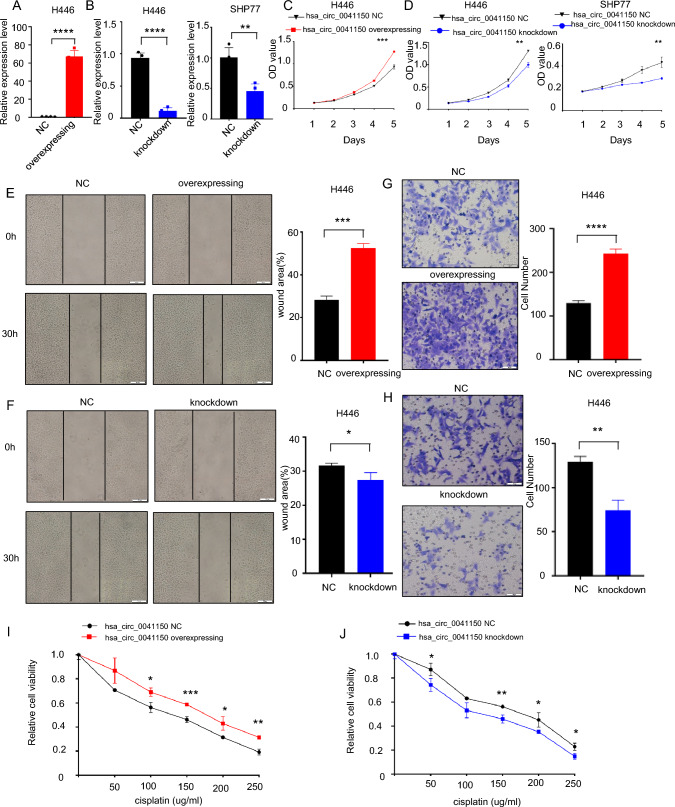
Promotion of hsa_circ_0041150 on the proliferation, invasion and migration of SCLC cells. **a** Detection of the overexpression efficiency of hsa_circ_0041150 in H446 cells, ****p < 0.0001. **b** Detection of the knockdown efficiency of hsa_circ_0041150 in H446 and SHP77 cells, ****p < 0.0001, **p < 0.01. **c** Detection of the proliferative activity of hsa_circ_0041150-overexpressing H446 cells by CCK-8 assay, ***p < 0.001. **d** Detection of the proliferative activity of hsa_circ_0041150 knockdown in H446 and SHP77 cells by CCK-8 assay, **p < 0.01. **e** Detection of the invasion ability of hsa_circ_0041150-overexpressing H446 cells by the scratch test, ***p < 0.001. **f** Detection of the invasion ability of hsa_circ_0041150 knockdown H446 cells by the scratch test, *p < 0.05. **g** Detection of the invasion ability of hsa_circ_0041150-overexpressing H446 cells by transwell assay, ****p < 0.0001. **h** Detection of the invasion ability of hsa_circ_0041150 knockdown H446 cells by transwell assay, **p < 0.01. **i** Detection of the cisplatin resistance ability of hsa_circ_0041150-overexpressing H446 cells by cisplatin resistance assay, *p < 0.05, **p < 0.01, ***p < 0.001. **j** Detection of the cisplatin resistance ability of hsa_circ_0041150 knockdown H446 cells by Cisplatin resistance assay, *p < 0.05, **p < 0.01

To investigate the role of hsa_circ_0041150 in the chemoresistance of SCLC, we established hsa_circ_0041150 stable overexpression cells in H446 cells (Fig. 6a), and hsa_circ_0041150 stable knockdown cells in both H446 and SHP77 cells (Fig. 6b). Cell proliferation assays revealed that hsa_circ_0041150 overexpression significantly increased the proliferation of SCLC cells (Fig. 6c), while knockdown of hsa_circ_0041150 resulted in the opposite effect (Fig. 6d). Furthermore, a cell scratch assay demonstrated that overexpressing hsa_circ_0041150 significantly enhanced the wound healing of SCLC cells (Fig. 6e), whereas knockdown of hsa_circ_0041150 slowed down the wound healing process (Fig. 6f). Additionally, the results of the transwell assay showed that overexpression of hsa_circ_0041150 significantly boosted the migration capacity of SCLC cells (Fig. 6g), whereas knockdown of hsa_circ_0041150 weakened the migration ability (Fig. 6h). Moreover, in the cisplatin resistance assay, we observed that overexpressing hsa_circ_0041150 remarkably strengthened the cisplatin resistance of SCLC cells (Fig. 6i), while knockdown of hsa_circ_0041150 led to a reduction in cisplatin resistance (Fig. 6j). Altogether, our findings indicate that hsa_circ_0041150 promotes the proliferation, invasion, migration, and cisplatin resistance of SCLC cells.

## Discussion

SCLC is considered the most aggressive subtype with the poorest prognosis among all lung cancer subtypes. While chemotherapy remains the primary treatment option, high metastasis and drug resistance pose significant challenges, resulting in a median survival time of only 4–5 months after chemotherapy. The first-line chemotherapy regimen for SCLC involves a combination of platinum-based drugs (cisplatin or carboplatin) and etoposide. Although platinum drugs have been widely used in the treatment of various tumors, long-term use has led to an increase in resistance. Multiple mechanisms of chemoresistance have been investigated, including DNA damage repair, autophagy, ATP-binding cassette transporters, cancer stem cells, epithelial-mesenchymal transition, and other related signaling pathways (Mu et al. 2021). Despite the unclear understanding of the transformation of drug-sensitive cells into drug-resistant cells during chemotherapy progression, there is an urgent need to identify a sample type for convenient repeat detection and essential biomarkers to detect drug resistance early in clinical settings.

In recent years, several studies have highlighted the role of circRNAs in either promoting or inhibiting the development of resistance to conventional chemotherapy in various tumors. These findings have opened up a new avenue for investigating antitumor drug resistance (Chen et al. 2022; Jiang et al. 2022). As the research on extracellular vesicles (EVs) advances, it has been revealed that EV circRNAs have significant clinical implications and value in the chemoresistance of malignant tumors (Wang et al. 2022a; Chen et al. 2023). Building on our previous research related to EVs (Chao et al. 2023), we aimed to identify differentially expressed circRNAs in EVs from platinum drug-resistant SCLC patients that could potentially serve as early indicative markers in clinical practice.

In our study, we collected clinical data from 54 patients diagnosed with SCLC and obtained serum samples at the time points of stable disease (SD) and progressive disease (PD) for each patient. We isolated EVs from the serum through hypercentrifugation and extracted RNA from both serum and serum EVs to assess the expression levels of six circRNAs. The results revealed a significant increase in the six circRNAs in serum when patients developed chemotherapy resistance and experienced clinical progression, with hsa_circ_0041150 showing remarkable promise. Notably, hsa_circ_0041150 demonstrated superior diagnostic value for drug resistance compared to traditional lung cancer-related tumor markers such as CA125, CA211, and ProGRP in SCLC, albeit slightly weaker than NSE. Furthermore, the expression levels of the six circRNAs in serum EVs also showed a substantial increase after platinum drug resistance, with hsa_circ_0041150 exhibiting exceptional diagnostic value for drug resistance, surpassing all traditional serum markers for lung cancer. Hence, serum EV circRNAs demonstrated consistency with the rising trend observed in serum and proved to be more effective biomarkers than serum circRNAs. This is in line with the findings of Kang et al. (2022), who reported that the diagnostic sensitivity and specificity of serum EV circRNAs were higher than those of serum circRNAs, suggesting the potential of serum EV circRNAs as a more effective biomarker for lung cancer.

With the rapid advancement of high-throughput transcriptome analysis techniques, liquid biopsy has emerged as a valuable tool in the comprehensive diagnosis and treatment of cancer, particularly due to its noninvasive nature, addressing the challenge of obtaining pathological tissue. Liquid biopsy enables the detection of specific markers in body fluids, and circRNAs have garnered attention for their stable and abundant presence in various body fluids, including plasma, serum, EVs, ascites, urine, and cerebrospinal fluid (Wang et al. 2021). In the context of lung cancer chemoresistance, current research on circRNAs in EVs primarily focuses on NSCLC. For instance, serum exosomal hsa_circ_0002130 has been found to promote osimertinib-resistance by sponging miR-498 (Ma et al 2020), tumor-derived exosomal circRNA_102481 contributes to EGFR-TKI resistance through the miR-30a-5p/ROR1 axis (Yang et al. 2021c), and exosome-transmitted circVMP1 facilitates cisplatin resistance in non-small cell lung cancer by targeting the miR-524-5p-METTL3/SOX2 axis (Xie et al. 2022). However, studies investigating EV circRNAs in SCLC remain limited, with only one study focusing on FLI1 exonic circular RNAs as a novel oncogenic driver promoting SCLC metastasis through the miR584-ROCK1 pathway (Li et al. 2019). To date, no study has explored the role of EV circRNAs in SCLC drug resistance. Therefore, our study aims to identify a novel biomarker for predicting platinoid drug resistance in SCLC, which could have significant implications for monitoring clinical efficacy.

In our study, we conducted dynamic monitoring of hsa_circ_0041150 levels in both serum and serum EVs of 21 SCLC patients who received a first-line chemotherapy regimen. At the initial diagnosis, prior to any clinical treatment, hsa_circ_0041150 levels were significantly higher in both serum and serum EVs compared to healthy subjects. Subsequently, following treatment with a platinum-based regimen, hsa_circ_0041150 levels decreased significantly, only to increase significantly again after the development of drug resistance. These findings suggest that hsa_circ_0041150 not only demonstrates diagnostic potential similar to other circRNAs (Xian et al. 2020), but it also allows for more dynamic monitoring of the entire treatment process. Our study also investigated the clinical association between hsa_circ_0041150 expression levels and various clinicopathological features. We observed that hsa_circ_0041150 levels were closely related to the time to progression from SD to PD, and SCLC patients with higher hsa_circ_0041150 levels in serum and serum EVs after drug resistance were more likely to experience chemotherapy resistance. This finding suggests that measuring hsa_circ_0041150 levels regularly during follow-up treatment holds great value in early detection of drug resistance and treatment-related changes.

In recent years, there has been growing attention towards understanding the role of circRNAs in cisplatin resistance in lung cancer. For instance, Shi et al. (2021) demonstrated that EV circ_0008928, derived from CDDP-resistant NSCLC serum, could reduce CDDP sensitivity and promote cell proliferation, migration, invasion, and glycolysis metabolism by modulating the miR-488/HK2 axis. Similarly, Shao et al. (2021) revealed that EV circ_PIP5K1A regulates the progression of NSCLC and cisplatin sensitivity through the miR-101/ABCC1 axis. However, the current research predominantly focuses on NSCLC, with limited exploration of the relationship between circRNAs and chemoresistance in SCLC. In our study, we observed that hsa_circ_0041150 played a stimulative role in promoting proliferation, invasion, migration and cisplatin resistance in SCLC cells. These findings align with the existing literature, which highlights the regulatory role of circRNAs in tumor proliferation, invasion, chemosensitivity, and other biological behaviors within the tumor microenvironment (TME) (Wang et al. 2022b). To explore the underlying mechanism, some studies have concentrated on the circRNA-miRNA-mRNA axis for regulation (Zhang et al. 2020; Zhou et al 2022), while others have investigated the interaction between specific circRNAs and their corresponding RBPs (RNA-binding proteins) (Okholm et al. 2020). As such, our subsequent research will focus on elucidating the mechanism by which hsa_circ_0041150 contributes to SCLC drug resistance.

Extracellular vesicles (EVs) play a crucial role in intercellular communication by delivering circRNAs to target cells, thereby influencing cancer development through various signaling pathways. Targeting EVs has emerged as a promising strategy for cancer treatment (Vahabi et al. 2022) and our study supports this notion, as we found that hsa_circ_0041150 present in tumor-derived EVs. In clinical settings, simultaneous monitoring of serum and serum EV hsa_circ_0041150 along with NSE (Neuron-Specific Enolase) can significantly enhance the sensitivity and specificity of diagnosing chemotherapy resistance in SCLC. However, we acknowledge that ultracentrifugation-based isolation of EVs, which identifies more EV biomarkers with higher concentrations, may not be widely accessible in clinical practice (Cao et al. 2019). Despite this limitation, our study demonstrates that the diagnostic efficiency of combining serum hsa_circ_0041150 with NSE surpasses that of all current serum detection indices, providing an alternative when EVs cannot be obtained through ultracentrifugation. As a result, our future research will focus on exploring the platinum resistance mechanism of hsa_circ_0041150 and the potential application of serum EV hsa_circ_0041150 as a biomarker using EV kits. This endeavour holds promise for enhancing the understanding of drug resistance mechanisms and facilitating the clinical use of hsa_circ_0041150 in SCLC management.

## Conclusions

In conclusion, our study provides evidence that hsa_circ_0041150 plays a significant role in promoting the proliferation, invasion, migration, and cisplatin resistance of SCLC cells. Furthermore, we highlight the potential of EV hsa_circ_0041150 as a valuable biomarker for monitoring chemotherapy resistance in small cell lung cancer patients undergoing first-line chemotherapy treatment.

## Supplementary Information

Below is the link to the electronic supplementary material.

Supplementary file1 (DOCX 81 kb)

## Data Availability

The NGS data for this study (GSE193854) can be found on the National Center for Biotechnology Information website. The datasets used and/or analyzed during the current study are available from the corresponding author upon reasonable request.
