# Supplementary material for: Hsa_circ_0041150 serves as a novel biomarker for monitoring chemotherapy resistance in small cell lung cancer patients treated with a first-line chemotherapy regimen
Source: J Cancer Res Clin Oncol. 2023 Aug 28;149(17):15365–82. doi: 10.1007/s00432-023-05317-6 (PMC10620281; doi:10.1007/s00432-023-05317-6)
Supplement: Supplementary file 1 — Supplementary file1 (DOCX 81 kb) [file 432_2023_5317_MOESM1_ESM.docx]

Table S1: The characterization of upregulated circRNAs

| **CircBase ID** | **Gene Symbol** | **Genomic Position** | **Length** | **Log_2_**  **(Fold change)** | **P-value** |
| --- | --- | --- | --- | --- | --- |
| hsa_circ_0041150 | RPH3AL | chr17:131559-177370 | 474 | 5.901 | 0.007 |
| hsa_circ_0017286 | SMYD3 | chr1:246021798-246027188 | 263 | 5.351 | 0.014 |
| hsa_circ_0040813 | BANP | chr16:88014642-88017865 | 292 | 5.152 | 0.028 |
| hsa_circ_0001187 | DOPEY2 | chr21:37619815-37620866 | 301 | 5.011 | 0.028 |
| hsa_circ_0003134 | PDGFRL | chr8:17446977-17447274 | 298 | 4.903 | 0.038 |
| hsa_circ_0000834 | C18orf1 | chr18:13387340-13387761 | 422 | 4.596 | 0.047 |
| hsa_circ_0000419 | RAB3IP | chr12:70193989-70195501 | 242 | 4.371 | 0.038 |
| hsa_circ_0083439 | MTUS1 | chr8:17532695-17542051 | 282 | 4.290 | 0.049 |

Table S2: The primers of upregulated circRNAs

| **circRNAs** | **Forward primer** | **Reverse primer** |
| --- | --- | --- |
| hsa_circ_0041150 | TGCAGTGAGCAAAGAGAGATGTG | GCAAACCCACTGATCATTCCC |
| hsa_circ_0017286 | GGCTTCCCGATATCAACATCT | CCAGTTAGCATATCAGCATCCTGT |
| hsa_circ_0040813 | TGCAACAAAGTGCGATGTTGT | GCGTTTCAAAGCAGGTTCGT |
| hsa_circ_0001187 | TCTGGAATGTTCTCAGAAAGGAG | TCCCTCTTGGAGGTTGTGTTT |
| hsa_circ_0003134 | TTTAAGGATTCTCGCCTCAGTTAC | TTGGGCTTCACCTTCTTGTTG |
| hsa_circ_0000834 | GATGGAAGTGACCTGAGCCTC | TGCTCCGCCTCTGAAACTGT |
| hsa_circ_0000419 | TAAGCATTGAACCAGTGGGATT | ATACAAGGATAAGTCAGCTTTGGTC |
| hsa_circ_0083439 | CCCTAGAATTCCTTGACAAAGGTT | TGTTTGTCCTGTGAATCGTTCC |

Table S3: The diagnostic efficiency of circRNAs and conventional tumor markers in SCLC chemoresistant patients

| Markers | AUC (95% CI) | Sensitivity(%) | Specificity（%） | P value |
| --- | --- | --- | --- | --- |
| hsa_circ_0017286 | 0.7047(0.6070,0.8025) | 75.93 | 57.41 | *0.0002 |
| hsa_circ_0040813 | 0.6603(0.5582,0.7625) | 50.00 | 75.93 | *0.0041 |
| hsa_circ_0001187 | 0.5766(0.4680,0.6853) | 55.56 | 62.96 | 0.1697 |
| hsa_circ_0003134 | 0.6235(0.5174,0.7295) | 59.26 | 66.67 | *0.0270 |
| hsa_circ_0000419 | 0.5811(0.4732,0.6890) | 46.30 | 70.37 | 0.1462 |
| serum EV hsa_circ_0017286 | 0.6884(0.5894,0.7875) | 61.11 | 64.81 | *0.0007 |
| serum EV hsa_circ_0040813 | 0.6566(0.5540,0.7591) | 53.70 | 66.67 | *0.0050 |
| serum EV hsa_circ_0001187 | 0.6804(0.5801,0.7807) | 68.52 | 61.11 | *0.0012 |
| serum EV hsa_circ_0003134 | 0.6480(0.5436,0.7524) | 55.56 | 72.22 | *0.0080 |
| serum EV hsa_circ_0000419 | 0.6140(0.5080,0.7200) | 55.56 | 66.67 | *0.0410 |
| CA125 | 0.6260(0.5209,0.7312) | 55.56 | 68.52 | *0.0239 |
| ProGRP | 0.6227(0.5115,0.7338) | 59.18 | 65.31 | *0.0364 |
| CA211 | 0.6159(0.5102,0.7217) | 68.52 | 51.85 | *0.0378 |

*P<0.05

Table S4 The sequences of hsa_circ_0041150 siRNA and shRNA

| si-h-hsa_circ_0041150-shRNA | sequences | complementary sequences |
| --- | --- | --- |
| 001(*) | AGTGAGCAAAGAGAGATGT | ACATCTCTCTTTGCTCACT |
| 002 | TGAGCAAAGAGAGATGTGA | TCACATCTCTCTTTGCTCA |
| 003 | CAAAGAGAGATGTGACTCC | GGAGTCACATCTCTCTTTG |

Fig S1 Significantlyy differentially expressed circRNAs (|log_2_FC|>4, p value<0.05) between H446 and SHP77 cells.

Fig S2 The diagnostic efficiency of circRNAs in serum and serum EVs compared with conventional tumor markers in chemotherapy resistant SCLC patients. **A** The diagnostic efficiency of serum circRNAs in chemoresistant SCLC patients. **B** The diagnostic efficiency of serum EV circRNAs in SCLC chemoresistant patients. **C** The diagnostic efficiency of conventional tumor markers in chemoresistant SCLC patients.
